# Evaluating major curriculum changes: Ensuring a psychologically safe learning environment is achieved for graduate students

**DOI:** 10.1002/ase.70144

**Published:** 2025-10-21

**Authors:** Gail Elliott, Grace Pinhal‐Enfield

**Affiliations:** ^1^ Department of Neuroscience and Cell Biology, Office of Education, Robert Wood Johnson Medical School Rutgers University Piscataway New Jersey USA; ^2^ Department of Neuroscience and Cell Biology, Anatomical Association, Robert Wood Johnson Medical School Rutgers University Piscataway New Jersey USA

**Keywords:** adult education, anatomical sciences/medical education, gross anatomy, team‐based learning

## Abstract

A graduate‐level master's gross anatomy course was recently rewritten to enhance delivery of its content in a manner that supports student learning. End‐of‐course evaluations from all 88 students showed highly favorable ratings for the curricular change, but a more detailed analysis is critical to determine whether a safe learning environment had been established. A safe learning environment is established where the curriculum is appropriately challenging, faculty are supportive, and students feel they belong. A measure of the success of a new course is typically the overall performance of the cohort, in addition to the student evaluations. One limitation of this is the use of Likert scores in student evaluations, which are universally devoid of nuanced information. In most instances, students are offered an opportunity to provide written feedback, and beyond a superficial read‐through of these comments, they are not typically analyzed for more information or with purpose. An AI‐based text analysis tool was used to undertake the underutilized technique of scoring the comments (sentiment data) to further investigate; the data were incorporated in this instance and highlighted several important categories that students deemed necessary to comment on, including: faculty availability, humor and knowledge, effective group dynamics, and a sense of belonging, among others. Based on the assessments of categories in the student‐written feedback, a safe learning environment was achieved.

## INTRODUCTION

In graduate‐level education, the student‐faculty dynamic is nuanced. Graduate students bring a higher level of social maturity than undergraduate students, having experienced both effective and ineffective teaching methods, which shapes their expectations for curriculum quality and the role of instructors. Many of these students are returning to academia after gaining real‐world experience through work or family responsibilities, influencing how they view the value of their education.^1^, ^2^ Their decision to pursue advanced qualifications, whether in fields like medicine, dentistry, or doctoral studies, is often driven by a desire to enhance their careers, income, and overall quality of life for themselves and their families. As these students, with diverse backgrounds and varying levels of ability, enter graduate and professional programs, courses designed to build academic credentials must accommodate this range of experience and skill. Consequently, it is essential to create a learning environment that supports all students and fosters a sense of safety and inclusion.

A psychologically “safe” learning environment meets several key criteria: it ensures students feel confident in the quality of their education, such as a curriculum that aligns with the specific standards (e.g., of a master's degree), and institutional support programs that enhance the student experience. This includes positive interactions with both peers and faculty. For instance, students should trust that the curriculum and its outcomes will meet the expectations set by course directors. This safe learning environment also fosters an atmosphere where students feel comfortable asking questions without fear of judgment by faculty and peers, and where they feel genuinely supported.^1^, ^2^ Additionally, students must believe in the rigor of the curriculum, knowing that the material is covered comprehensively and assessed fairly. Unfortunately, since the pandemic, the learning environment has deteriorated in some respects. Contributing factors include student reluctance to engage in‐person, faculty burnout from online teaching causing them to struggle to create effective communication pathways with students, and mutual feelings of isolation and complaints about unreasonable expectations, all of which have created a more negative learning atmosphere.^1^, ^2^, ^3^, ^4^

The learning environment has been extensively studied over the past few decades, with many studies recognizing its fundamental role in student motivation and progression. These studies have largely focused on assessing students' perceptions of a positive learning environment, the involvement of faculty, and the factors that contribute to such an environment, while also considering feedback from both students and faculty. A consistent category across these studies is the students' perspective on what constitutes a positive learning environment.

Moos^5^, ^6^ identified three key components of the learning environment^1^: the institution, including the curriculum and support programs,^2^ faculty, focusing on their attitudes, teaching approaches, and willingness to engage with students, and^3^ the students' ability to master the material and achieve the learning outcomes set by the faculty. Within each of these areas, students frequently commented on common categories. For example, they often highlighted the engaging nature of faculty humor, their willingness to answer questions, and their availability for meetings. Additionally, students frequently mentioned the manageability of the course material and the ability to meet expectations as key factors influencing the learning environment. Moos^5^, ^6^ also pioneered a novel approach to evaluating learning environments by scoring students' comments on these specific areas. This method is particularly effective because it allows educators to evaluate open‐ended student feedback, going beyond traditional Likert‐scale assessments, which limit student responses. Analyzing these comments helps faculty better understand students' perceptions of the learning environment and assess whether a safe and supportive atmosphere has been established. This framework described by Moos has been validated^1^ and so was the favored approach chosen for this endeavor.

This manuscript examines changes to a graduate‐level learning environment following a major curriculum overhaul, aimed at better aligning with the abilities of the students admitted. While a curriculum change alone does not guarantee a positive learning environment, it is a crucial component of creating one. The focus of this manuscript is on evaluating student feedback to determine whether a “safe” learning environment has been successfully achieved, where a safe learning environment would be confirmed by high Likert scores (4+) and feedback through written comments (sentiment scores) correlating to the high end of the Likert scale.

## METHODS

An IRB approval (IRB #: Pro2024000498) was obtained from the Human Research Protection Program, Office for Research, Rutgers University, to assess student evaluations from the 2024 cohort of the 13‐week graduate‐level gross anatomy course. The curriculum was redesigned after it was determined that student performance in previous years was concerningly low, largely due to a mismatch between student abilities and curricular expectations. A total of 88 students (full cohort, 100%) provided feedback for the course, in accordance with IRB standards and compliance.

The graduate program in Biomedical Science at Rutgers University School of Graduate Studies offers a 13‐week anatomy course designed for students pursuing professional degrees such as medicine. Many of these students work full‐time, some have been away from formal education for a while, and others may not have initially met the academic requirements for medical school. As a result, the course serves a diverse range of academic abilities, necessitating curricular adjustments to establish a baseline for graduate‐level anatomy learning. The original course covered the back, upper limbs, thorax, abdomen, and pelvis over 13 weeks. It was a traditional didactic course, with no team‐based learning (TBL), and most grading was based on two exams: a midterm and a final. However, prior iterations of the course revealed a mismatch between student abilities and the curriculum's expectations. To better support a diverse student body, the course was redesigned to incorporate TBL, biweekly progress quizzes, and increased faculty support. As a result, the course was redesigned to better accommodate a wider range of learning abilities, where learning abilities in this context are an individual's capacity to acquire, process, understand, and apply new knowledge and skills based on experience or instruction.

This study applies Moos'^5^, ^6^ framework, which has been validated in prior research,^1^ to analyze the learning environment by scoring students' comments in addition to traditional Likert scale assessments. Open‐ended feedback provides a richer understanding of students' perceptions of faculty support, course manageability, and peer dynamics. This approach of scoring open‐ended comments (sentiment data) using an AI‐based text analysis tool, in addition to evaluating Likert scale scores, allows for a more nuanced and robust investigation into student feedback and the development of a safe learning environment. The limitation of relying solely on Likert scores is that they constrain the range of possible responses, potentially overlooking important nuances in students' feedback. Further limitations to using Likert data are also in not being sure how to correctly analyze it, whether the midpoint on the scale is useful, while students responding to evaluations may deliberately always choose the most neutral option (midpoint).^7^, ^8^ Despite many of the challenges surrounding the use of Likert scales, their universal use continues and is widely accepted in most university student evaluations. In many of these types of evaluations, students are able to provide written feedback, but most institutions do not tend to analyze this sentiment data for trends, only looking at it to confirm what the Likert score is showing.

The survey included 28 questions designed to capture students' overall perceptions of the course content, faculty availability and approachability, the incorporation of new materials, and the course's emphasis on spaced active learning and holistic, team‐based approaches. Each question featured a Likert scale (5 = Strongly Agree, 4 = Somewhat Agree, 3 = Neither Agree nor Disagree, 2 = Somewhat Disagree, 1 = Strongly Disagree) along with open‐ended prompts for students to provide additional written feedback (sentiment data) about components of the course that worked and did not work. The survey was significantly shortened from 116 questions to just 28. To encourage participation, students were given the opportunity to complete it while waiting in class between exams—an approach not used in previous years. The open‐ended questions were carefully worded to avoid bias and encourage honest, constructive feedback. For example, students were asked, “*What could be improved? What suggestions do you have for future implementations of this course?*” and “*Please comment on how the various team‐based activities (lab, TBLs, group exams) affected your future participation and attitude toward teamwork*.” The goal was to gather transparent, useful insights to enhance the course environment for both current and future students.

To ensure clarity in the classification process, student comments were analyzed to identify key phrases and words that corresponded to specific subcategories within the broader categories defined in Table 2. For example, the statement, “*Her sense of humor expressed in every means…*” was categorized under “Knowledgeable and Humorous” based on phrases such as “humor” and “knew how to deliver a lecture.” Similarly, the phrase “approachable” was considered for categorization under “Emotional Support,” but due to its context within the comment, it did not meet the criteria for that classification. This approach ensured that categorizations were based on explicit textual evidence while maintaining clear distinctions between categories. The decision to group “Knowledgeable and Humorous” as a single category was based on the frequency with which students linked these attributes in their comments. Rather than viewing humor as an isolated trait, students often described it as an integral component of faculty effectiveness, enhancing engagement and knowledge retention. This justified the combination of these traits into unified subcategories.

For the data analysis, the approach taken was to carry out conventional content analysis by deductively assigning coding to the data as the analysis was being carried out.^8^ This was based on the work by Moos^5^, ^6^ where major categories in the data were initially set for the assessment because these components are key to the identification of a psychologically safe learning environment. However, the subcategories under each of the major categories were inductively identified during the data analysis using an AI‐based text analysis tool to identify subcategories of these major categories. For AI‐based text analysis, we used ChatGPT 4.0, which is a publicly available large language model developed by OpenAI. We ensured that the tool's use was consistent with ethical guidelines (closed system, de‐identified data). For data input into ChatGPT, the categories for the model of Moos were established first and then a review of the subcategories by the authors was determined by manually reading through the data. The AI tool was then asked to confirm/verify these subcategories and to summarize comments that held these categories in them. It was also asked to locate these subcategories to see if any further patterns were located, and none were identified. This approach allowed for a deeper investigation of the data and attempts to reduce any bias that would be introduced by identifying these subcategories ahead of time (see Table 2 for the major categories and subcategories for each). All of the data analysis and identification of subcategories were carried out by the primary author. The exact prompts provided to ChatGPT are included in the online Supporting Information for transparency of the AI analysis process.

## RESULTS

The AI‐based text analysis tool revealed several key categories derived from student feedback. Table 1 summarizes the Likert scale survey results, which provide a quantitative overview of students' perceptions of course quality, organization, and team‐based activities. To complement these quantitative findings, we conducted an AI‐assisted content analysis of open‐ended student comments. The resulting categories and subcategories are presented in Table 2.

**TABLE 1:** Likert scale feedback for questions and breakdown of student numbers for each from the student evaluation (where 1 = Strongly Disagree, 2 = Somewhat Disagree, 3 = Neutral, 4 = Somewhat Agree, and 5 = Strongly Agree).

Question asked	Overall Likert score	No. (%) of students responding				
		5	4	3	2	1
Do you agree overall the course quality and effectiveness was expected for a Master's level program?	4.94	75 (94%)	5 (6%)	0 (0%)	0 (0%)	0 (0%)
Did exam questions reflect the topics emphasized?	4.90	72 (90%)	8 (10%)	0 (0%)	0 (0%)	0 (0%)
Was the course well‐organized overall?	4.84	67 (84%)	13 (16%)	0 (0%)	0 (0%)	0 (0%)
Were the laboratory sessions effective in helping me learn the material?	4.80	66 (82%)	12 (15%)	2 (3%)	0 (0%)	0 (0%)
Were the four team‐based learning activities (TBLs) helpful toward your overall mastery of the content?	4.71	62 (78%)	12 (15%)	4 (6%)	1 (1%)	0 (0%)
Were the team‐based exams helpful in guiding you to understand concepts you missed in the exams?	4.72	64 (81%)	9 (11%)	5 (6%)	1 (2%)	0 (0%)
Did your biweekly preparation for the cumulative progress quizzes help you to stay up‐to‐date and prepare more effectively for exams?	4.80	65 (82%)	12 (15%)	2 (3%)	0 (0%)	0 (0%)
Average Likert score	4.82					

**TABLE 2:** The subcategories identified from student comments (sentiment data) are shown in relation to the three major categories Moos^5^, ^6^ identifies as essential to a psychologically safe learning environment. The % of the total number of comments that contribute to each of the subcategories is also shown.

Faculty and TAs (% of all comments)	Individual and peers (% of all comments)	Institution and course (% of all comments)
Available and responsive (28%)	Effective group dynamics (25%)	Instructional materials (32.5%)
Emotional support (10%)	Inclusive learning culture (20%)	Scheduling and accountability (20%)
Knowledgeable and humorous (6%)	Sense of belonging (15%)	Human donor lab experience and access (27.5%)

*Note*: The % does not align to 100% because many students commented on multiple of these different subcategories in one comment.

### Overall Likert score feedback

The Likert scale data from Table 1 consistently show that students rated various aspects of the course highly, with most responses falling within the 4–5 range (somewhat agree to strongly agree). For example, of the students, 94% strongly agreed and 6% somewhat agreed that the course quality and effectiveness met the expectations of a master's level program, while 90% strongly agreed and 10% agreed that the exam questions accurately reflected emphasized topics. Additionally, 84% strongly agreed and 16% somewhat agreed that the course was well‐organized, and 82% strongly agreed while 15% somewhat agreed about laboratory sessions effectively supporting their learning. These high scores suggest that the course structure, faculty engagement, and collaborative learning activities successfully fostered a positive learning environment.

The data also indicate that TBL activities and collaborative assessment methods contributed to student engagement. The four TBL activities received a high approval rating, with 78% of students strongly agreeing and 15% somewhat agreeing that these activities supported content mastery. Similarly, 81% of students strongly agreed and 11% somewhat agreed that the team‐based exams were helpful in reinforcing concepts they had missed.

While Likert scale data provide a valuable quantitative overview, they do not fully capture the nuances of student experiences. To gain deeper insight into the factors contributing to a safe and inclusive learning environment, sentiment data analysis was performed on student comments (Table 2). This qualitative approach revealed key themes, such as faculty availability, humor, group dynamics, and a sense of belonging, all of which contributed to positive student perceptions.

### Faculty and teaching assistants (TAs)

Students highlighted the availability and responsiveness of faculty, the emotional support they received, and the knowledgeable and humorous delivery. The impact of the four teaching faculty and three teaching assistants (TAs) was one of the highlights of the course through the feedback, with an average Likert score of 4.87. Students consistently reported faculty were supportive, available, kind, knowledgeable, and patient. Students particularly focused on clear explanations, incorporation of humor, and real‐world clinical correlations. For example, the below comments were very typically threaded throughout the evaluation feedback:

I really appreciate the way Dr. X lectures, and I think having questions interspersed between lecture slides is really helpful in connecting material to potential exam questions.

Her sense of humor expressed in every means of communication, whether they be lectures, emails, or in‐person announcements, made the whole experience with the course very pleasurable. In addition, she was very approachable and knew how to deliver the lecture content well, and all of those synergistically worked in a way that made me love the course.

There were also consistently appreciative comments for faculty willingness to help through office hours, TA review sessions, and one‐to‐one support. The TA‐led review sessions held once a week were considered very highly in helping students to deepen their understanding of the structures and to learn useful mnemonics. Students also consistently emphasized their appreciation for prompt responses to questions during lab sessions and through email, as well as the detailed nature of the email communications. Finally, there were multiple comments talking about the emotional support, encouragement and morale‐boosting provided by the faculty in encouraging students when they were struggling and taking the time to re‐explain topics in different, more manageable ways in a positive manner that improved student confidence. There were also multiple comments about the faculty making the effort to check in on student well‐being.

Most importantly, there were no comments about faculty that would raise concern or were negative about their support or attitude toward students. Rather, the comments were overwhelmingly favorable.

### Individual and peer experiences

In this particular area, students commented on three specific subcategories: the benefits of working in groups/teams, an inclusive learning environment, and a sense of belonging. Many of the students had not experienced team work before, so this aspect as a strong sub‐category was not surprising (Likert score for TBLs = 4.71). There were also several components of the course including TBLs, human donor dissection labs, and group exams that required students to work in groups and they maintained these groups for the entire course, giving them a chance to get to know each other. Most of the comments about the teams were consistent as students expressed that they had to prepare better for the sessions so they did not let their group down, and found it very beneficial to learn from each other's perspectives. Most also acknowledge that they formed bonds with their peers and that these groups also became study groups outside of the required sessions. For example, the comment below reflects the nature of most of the comments regarding the team work:

I thought the various TBLs were useful especially for digesting and putting together material. Also gave us the ability to get to know our group members even more which is always helpful.

Some of the students also commented on their initial reservations about being part of the group, but their experiences within the course led them to comment on the value of the experience and being open to future teamwork experiences:

I used to be very hesitant with group work for many reasons. The TBLs completely changed my attitude towards group learning. I now see how beneficial it can be to collaborate and learn together.

A second aspect that students discussed involved the collegial work environment that was developed, where they felt safe to ask questions or to make mistakes. There was also an acknowledgment that the humor among faculty and TAs was instrumental in making them seem more accessible, the environment more collegial, and the course itself more enjoyable. This tied in closely with acknowledgment that faculty regularly reinforced the importance of student well‐being along with the academic success and this balanced and realistic approach seems to have reduced stress and motivated students to engage better with the material.

Finally, students commented on a sense of belonging because the amount and difficulty of the course material were manageable. The team activities that encourage interactions with peers also helped the students make connections with their peers and eased the transition into the master's degree that this course is a part of. There were even several comments touching upon the fact that if this course were the first course in the curriculum, the students would enjoy the program more, as shown in the comment below:

Keep it as it is. It is great. It would be very helpful, if this course would be the first one taken in the master program, as it helps with accommodating to this new way of studying, and with making new friends.

There were no concerns or negative comments in this area, rather several requests for more TBL activities and opportunities for further engagement in the human donor lab.

### Institution and course

Three major subcategories for the institution and course, including the course structure and organization, scheduling and accountability, and access to the human donor laboratory, were identified from the sentiment data. There was wide agreement among the cohort that the materials and course requirements were at a master's degree level (Likert score = 4.94). The major commentary here focused on the course structure and the careful integration and complementation of various components (e.g., TBLs, lectures, human donor labs). There was also appreciation for the diversity of assessment methods, including progress quizzes that helped students stay up‐to‐date with the material and their learning. A few students also commented on how much they appreciated that something, however small, was planned for each day to keep them actively engaged and on pace, preventing poor learning practices such as last‐minute cramming for exams.

Students also commented on their appreciation for the 24/7 access to the donor lab for studying purposes and appreciated detailed materials, such as the in‐house lab manuals, lecture slides, and supplemental videos. However, there were some minor comments that further human donor images in the in‐house lab dissection guides were preferable with more initial instruction from the faculty for each dissection lab. Several students also expressed a dislike for the self‐directed study approach that was gently incorporated with required pre‐reading of the lab manual ahead of lab, which preceded the related lecture. This approach emphasized a preference for the lab to precede the associated lecture and put an emphasis on students reading ahead of the associated activity. Finally, students requested more review sessions from the TAs within the course, highlighting their value in preparing for exams and staying up‐to‐date.

## DISCUSSION

Evaluating sentiment data from the course feedback was an enlightening endeavor because it offered insight into aspects of teaching that students consider important and allowed for a nuanced approach to assessing the success of the new course curriculum. However, before delving into the sentiment data, it is essential to first examine the Likert scale data presented in Table 1, as it provides a broad, quantitative confirmation of a positive and inclusive learning environment.

These findings align with research indicating that an inclusive learning environment is characterized by structured engagement, faculty support, and opportunities for peer collaboration.^5^, ^6^ The consistently high ratings across different course components support the conclusion that students felt both included and supported within this learning framework. Further, evidence for student acknowledgment of support is seen through student engagement, which has notably increased. In prior years, only ~26 students completed the course evaluation, whereas in the first year following curricular change, 88 students (the full cohort) participated. In addition to reducing question numbers in the survey from 116 to 28 and providing time to fill in the evaluation between exams, this participation may also reflect improved student investment and satisfaction with the course structure.

One of the strongest categories that emerged from the sentiment analysis was the faculty's role in shaping the learning environment. Students consistently praised faculty for being available, responsive, and supportive, with many emphasizing the positive impact of faculty humor and encouragement. This aligns with the high Likert scores for course organization and faculty engagement, reinforcing the conclusion that instructor involvement played an essential role in creating a safe and inclusive space for learning. Additionally, students highlighted the effectiveness of team‐based activities, which helped foster a sense of belonging and collaborative learning. While some initially expressed reservations about group work, many later acknowledged the benefits of working closely with peers and forming study groups. These sentiments correspond with the high Likert scores for TBLs and team‐based exams, illustrating how structured group activities contributed to an inclusive learning culture.

Institutional factors, such as course structure, scheduling, and access to resources were also key contributors to student satisfaction as described in the framework for a positive learning environment by Moos.^5^, ^6^ Many students appreciated the availability of the human donor lab and the structured, cumulative nature of assessments, which kept them engaged throughout the course. However, some students expressed a preference for additional faculty‐led review sessions and a more structured approach to lab‐based learning.

By integrating both Likert scale data and sentiment analysis, this study provides a comprehensive assessment of the learning environment. The high Likert scores confirm that students found the course to be well‐organized, inclusive, and supportive, while the sentiment data offer deeper context on the specific elements that contributed to this perception. This dual approach underscores the importance of structured faculty support, collaborative learning opportunities, and thoughtful course design in fostering a safe and engaging graduate education experience.

## STUDY LIMITATIONS

There are several limitations to this study. Selection bias may have occurred where students with stronger opinions were more likely to leave comments, potentially skewing the data, even if they represent a smaller portion of respondents. Additionally, some students may have provided overly positive feedback due to perceived expectations or uncertainty about the anonymity of evaluations. Knowing that faculty were actively revising the curriculum to support their academic progress, students may feel compelled to offer more favorable comments than they otherwise would.

Likert scores also present challenges whenever they are assessed, particularly due to the inclusion of a neutral response option, which may dilute meaningful insights from more opinionated students. Furthermore, sentiment analysis relies on subjective interpretation, and nuanced responses may be overlooked as broader trends are identified in the data. Finally, as a single‐cohort study, the long‐term viability of the observed safe learning environment remains uncertain. Future iterations of the course will determine whether these positive outcomes persist over time or if they are primarily a short‐term effect of the curriculum rewrite and faculty's transparent communication, which may have generated goodwill and influenced students to provide more favorable feedback.

## CONCLUSION

Based on the assessments of course feedback, a psychologically safe learning environment was perceived by students in a revamped course that offered more faculty availability, humor and knowledge, effective group dynamics, and a sense of belonging, among others. Educators intending to revamp curricula should consider framing their changes around the pillars of the learning environment, as detailed by Moos.^5^, ^6^ Finally, while there were limitations to this study, identifying categories in student sentiment feedback offered an opportunity to gain a greater understanding of the nuance that exists within the framework of Moos, such as humor within the faculty–student dynamic that contributes to the development of a positive learning experience.

## AUTHOR CONTRIBUTIONS

**Gail Elliott:** Conceptualization; writing – original draft; methodology. **Grace Pinhal‐Enfield:** Writing – review and editing; methodology; conceptualization.

## CONFLICT OF INTEREST STATEMENT

The authors have no competing interests to declare that are relevant to the content of this article.

## Supporting information


Data S1.
