# Supplementary material for: Evaluating major curriculum changes: Ensuring a psychologically safe learning environment is achieved for graduate students
Source: Anat Sci Educ. 2025 Oct 21;19(1):93–9. doi: 10.1002/ase.70144 (PMC12748034; doi:10.1002/ase.70144)
Supplement: Supplementary file 1 — Data S1. [file ASE-19-93-s001.docx]

**Supplementary Information**

**Manuscript Title:***Evaluating Major Curriculum Changes: Ensuring a Psychologically Safe Learning Environment for Graduate Students*

**Authors:** Gail Elliott, PhD, AFHEA; Grace Pinhal-Enfield, PhD, ACUE

**Supplementary Appendix: Transparency of AI Analysis Process**

### **Purpose of AI Use**

An AI-based text analysis tool (ChatGPT, OpenAI, version 4.0) was used to assist with the categorization and summarization of student written feedback. The AI was not used to generate raw data. Instead, it supported the verification of categories derived from Moos’ framework and the summarization of sentiment across responses.

### **Prompts Provided**

Representative prompts given to ChatGPT are included below. All data entered were fully de-identified prior to analysis.

1. **Category Verification** *Prompt:*“Using the following predetermined categories from Moos (Faculty & TAs, Individual & Peers, Institution & Course), review this set of de-identified student comments. Confirm whether the proposed sub-categories (e.g., Availability & Responsiveness, Sense of Belonging, Instructional Materials) are consistent with the data. Suggest refinements if necessary.”
2. **Sentiment Summarization** *Prompt:* “Summarize the following de-identified student comments into concise statements that reflect their main sentiment (positive, neutral, or negative).”
3. **Pattern Identification** *Prompt:* “Identify whether any additional sub-categories beyond those already proposed emerge from this set of comments. If none are evident, confirm that the current sub-categories are sufficient.”

**Validation Process**

- The primary author conducted a manual review and coding of all student comments.
- AI outputs (verification of categories, sub-category summaries, sentiment ratings) were cross-checked against manual coding.
- Any discrepancies were resolved by the authors, with manual interpretation taking precedence.
- No new sub-categories were added solely on the basis of AI output.

**Ethical Safeguards**

- Student evaluation data were de-identified before input.
- Analysis was performed in compliance with IRB approval (Pro2024000498).
- No identifiable information was used, and no further training of the AI model occurred.
